# Randomized Controlled Trials of Tianma Gouteng Decoction Combined with Nifedipine in the Treatment of Primary Hypertension: A Systematic Review and Meta-Analysis

**DOI:** 10.1155/2020/5759083

**Published:** 2020-02-07

**Authors:** Jia Tai, Junbo Zou, Xiaofei Zhang, Yu Wang, Yulin Liang, Dongyan Guo, Mei Wang, Chunli Cui, Jing Wang, Jiangxue Cheng, Yajun Shi

**Affiliations:** Shaanxi Province Key Laboratory of New Drugs and Chinese Medicine Foundation Research, Pharmacy College, Shaanxi University of Chinese Medicine, Xianyang, 712046, China

## Abstract

**Background:**

Hypertension is a primary risk factor for cardiovascular disease (CVD). Tianma Gouteng decoction (TGD), originating from Zabingzhengzhixinyi, has been used for thousands of years in China to treat hypertension, giddiness, and migraine. This updated meta-analysis aimed at assessing the efficacy and safety of TGD combined with nifedipine in the treatment of primary hypertension.

**Methods:**

Related research published prior to September 1, 2019, was found in electronic databases without language limitations. Fourteen studies were selected and analyzed for specified criteria, including the quality of the studies. All outcomes were recorded exhaustive. Data management and analysis were performed using RevMan 5.3 software.

**Results:**

A total of 1,537 (769 cases in the experimental group and 768 cases in the control group) patients were enrolled. The total efficacy rate was improved significantly for the combination of nifedipine with TGD compared to nifedipine treatment alone (*I*^2^ = 22%, RR = 1.17, and 95% CI: 1.12 to 1.22). Traditional Chinese medicine (TCM) symptoms of patients were obviously improved in the experimental group than in the control group (*I*^2^ = 22%, RR = 1.17, and 95% CI: 1.12 to 1.22). Traditional Chinese medicine (TCM) symptoms of patients were obviously improved in the experimental group than in the control group (*I*^2^ = 22%, RR = 1.17, and 95% CI: 1.12 to 1.22). Traditional Chinese medicine (TCM) symptoms of patients were obviously improved in the experimental group than in the control group (*I*^2^ = 22%, RR = 1.17, and 95% CI: 1.12 to 1.22). Traditional Chinese medicine (TCM) symptoms of patients were obviously improved in the experimental group than in the control group (*I*^2^ = 22%, RR = 1.17, and 95% CI: 1.12 to 1.22). Traditional Chinese medicine (TCM) symptoms of patients were obviously improved in the experimental group than in the control group (*P* < 0.00001) when two studies (shicaihong 2017 and xiaoyugao 2017) were removed. And the results of DBP showed no heterogeneity (*I*^2^ = 22%, RR = 1.17, and 95% CI: 1.12 to 1.22). Traditional Chinese medicine (TCM) symptoms of patients were obviously improved in the experimental group than in the control group (*P* < 0.00001) when two studies (shicaihong 2017 and xiaoyugao 2017) were removed. And the results of DBP showed no heterogeneity (

**Conclusion:**

The combination of TGD and nifedipine has a better effect in the treatment of hypertension, including blood pressure lowering and patients' TCMs improving. However, our findings must be handled with care because of the small sample size and low quality of clinic trials cited. Other rigorous and large-scale RCTs are in need to confirm these results.

## 1. Background

Hypertension is an important risk factor for cardiovascular disease (CVD) worldwide, and it is also a major risk factor for stroke and coronary heart disease (CHD) in China [1, 2]. As a calcium channel blocker, nifedipine is employed to broadly treat hypertension in clinics [3]. However, some adverse reactions (AEs) to nifedipine are common, such as edema, rash, headache, and dizziness [4–6]; it can even cause serious adverse reactions within the cardiovascular system, as stated in the drug warnings for nifedipine.

Combination therapy, which is considered to be beneficial for enhancing the antihypertensive effect without increasing AEs [2], is the basic principle for treating hypertension in the Guide to Prevention and Treatment of Hypertension (version 2010). The combination of Chinese and Western medicine is a prevalent therapeutic regimen for treating numerous forms of disease in China. Tianma Gouteng decoction (TGD), originating from Zabingzhengzhixinyi, is a classic traditional Chinese medicine (TCM) prescription used for thousands of years in China for the treatment of hypertension, giddiness, and migraine [7, 8]. TGD is composed of *Uncaria rhynchophylla* (Miq.) Jacks., *Gastrodia elata* Bl., *Scutellaria baicalensis* Georgi, *Eucommia ulmoides* Oliv, *Radix cyathulae*, *Loranthus parasiticus*, abalone shell (the abalone shell can be collected without the animal being harmed in any way), Gardenia, *Leonurus japonicus*, Caulis polygoni multiflori, and Poria cocos, all of which are standard in the Chinese Pharmacopoeia 2015 edition [7].

Several meta-analyses [9–12] have been conducted on the use of TGD to treat primary hypertension focusing on TGD therapy alone. However, the combined use of TCM with Western medicine has been proven to be more effective for many diseases by an increasing number of evidence-based practices. A meta-analysis reported that TGD combined with nifedipine had better effects than nifedipine alone in the treatment of primary hypertension, but the consequence measures were not exactly sufficient [13]. Here, we provided an updated and expanded meta-analysis with timely clinical studies that were mainly conducted between 2014 and 2019. Moreover, the measurements indicated in our paper are more comprehensive than previous reports.

## 2. Methods

### 2.1. Search Strategy and Selection Criteria

We searched CNKI, PubMed, VIP, EMBASE, Wanfang, Cochrane Library, and CBM. To conduct a comprehensive search, studies published prior to September 1, 2019, were investigated without language limitations. The search terms used were as follows: “Tianma Gouteng decoction” and “hypertension” or “nifedipine” and “hypertension.” All corresponding articles were downloaded into Endnote software (version X8, Thomson Reuters, Inc., New York, USA) for further investigation.

### 2.2. Inclusion and Exclusion Criteria

The inclusion criteria were designed according to the suggestions of doctors, as follows: patients diagnosed as having primary hypertension by meeting the criteria of Guide to Prevention and Treatment of Hypertension 2010, Guiding Principles for Clinical Research of New Drugs in Traditional Chinese Medicine, Chinese Medicine Dialectical Diagnosis Efficacy Standard, Chinese Medicine Diagnosis and Treatment of Heart Disease Efficacy Standards and Norms, or Guide to Prevention and Treatment of Hypertension in China. Studies were presented as randomized control trials (RCTs). The intervention used for patients was TGD combined with nifedipine in the experimental group and only nifedipine in the control group. The measurement of the outcome of each article must have contained a total antihypertensive efficacy. The following indices in the articles must contain at least one of the following: blood pressure, TCMs, serum creatinine, adverse events, and blood urea nitrogen.

The following criteria were utilized to exclude conditions: (1) nonrandomized controlled trials; (2) secondary hypertension; (3) hypertension and other illnesses; (4) patients received drugs other than TGD and nifedipine in RCTs; and (5) studies such as reviews, animal experiments, and case report that were considered to be irrelevant to the theme.

Patients that had systolic blood pressure (SBP) greater than or equal to 140 mmHg and/or diastolic blood pressure (DBP) greater than or equal to 90 mmHg were diagnosed with hypertension in the included studies. To measure the diagnostic efficacy of antihypertension treatment, the total efficiency is equal to significant effect and effective summation. Treatments were considered significantly effective when DBP returned to normal levels and reduced by at least 10 mmHg, or DBP did not return to normal levels, but the reduction was at least by 20 mmHg. The treatment was considered effective when DBP returned to normal levels and decreased by less than 10 mmHg, when DBP did not return to normal levels, but the reduction was by 10 mmHg∼19 mmHg, or there was a reduction in systolic blood pressure of at least 30 mmHg. Treatment was considered invalid when DBP and SBP did not change significantly or even got worse.

TCM symptoms of hypertension criteria were as follows: (1) significant effect: obvious improvement in clinical symptoms, (2) effective: clinical symptoms slightly improved, and (3) invalid: symptoms and signs have no significant changes or even worse.

### 2.3. Assessment of Trial Quality

In order to assess the risk of bias, three authors (Jia Tai, Junbo Zou, and Yu Wang) independently evaluated the study validity according to the Cochrane Handbook for Systematic Reviews of Interventions [14].

Six criteria assessing bias and quality were evaluated according to whether the articles described the following:

1. Random sequence generation (selection bias)
2. Allocation concealment (selection bias)
3. Blinding of participants and personnel (performance bias)
4. Blinding of outcome assessment (detection bias)
5. Incomplete outcome data (attrition bias)
6. Selective reporting (reporting bias) and other biases

Three levels were used to assess each checklist item. “Low risk” of bias suggested that the program was sufficient. “High risk” of bias indicated that the description of methods or treatment program was not sufficient enough or was abnormal. “Unclear risk” of bias indicated that there were no descriptions of methods or the treatment program. Any objections among the evaluators (Jia Tai, Junbo Zou, and Yu Wang) were determined through conversation with a fourth author (Yulin Liang).

### 2.4. Data Extraction

Information from the articles selected in this study included authors, year of publication, number of primary hypertension cases in the experiment and control groups, gender and age of patients, treatment period, random method, interventions, and evaluation standard, and the evaluation indexes were independently extracted by the three authors (Jia Tai, Junbo Zou, and Yu Wang). This information is provided and arranged in Tables 1 and 2.

### 2.5. Statistical Analysis

A Cochrane collaboration meta-analysis review methodology was applied in this study, with Review Manager 5.3 (Cochrane Collaboration) used to perform statistical analysis. The heterogeneity of the studies was determined by *I*^2^ tests and Q statistics. If the data had low heterogeneity (*P* ≥ 0.1 and *I*^2^ ≤ 50%), a fixed-effects model was applied. If the data had high heterogeneity (*P* < 0.1 or *I*^2^ > 50%), a random-effects model was applied. Latent issue bias was shown by funnel plots. Index measures, such as antihypertensive efficacy and TCMs, were thought to have dichotomous variables and evaluated by risk ratio (RR) with 95% confidence intervals (CIs). Continuous variables (such as BP) were rated by the mean difference (MD) with 95% confidence intervals. The significance of RR or MD was analyzed by a *z*-test, and *P* < 0.05 was considered to be indicative of statistical significance. The potential publication bias was assessed by constructing funnel plots.

## 3. Results

### 3.1. Description of Studies

**Figure 1:**
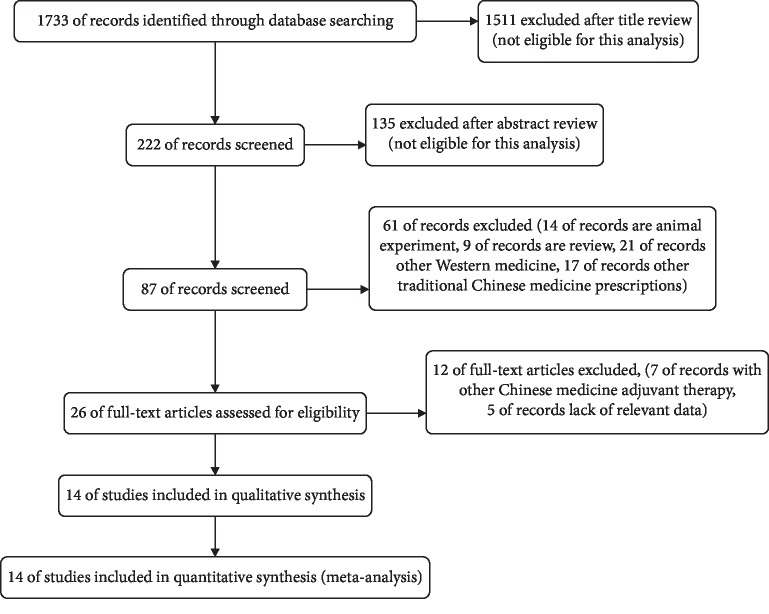
Processing of the studies extracted for the meta-analysis.

Studies took place between 2012 and 2017 (Table 1); all were RCTs. A total of 1,733 potentially corresponding studies were identified by our primary search, and 132 articles were exempted for repeat. We excluded 1,514 studies because they obviously did not meet the theme of this paper. Then, a full-text review was conducted on the remaining 87 articles. A total of 73 studies were exempt for the following reasons: 23 studies were animal experiments, 14 articles had vague diagnoses, and 36 articles referred to different intervention methods. Fourteen [15–21, 23–29] studies had adequate index data to permit the calculation of effect sizes for inclusion in this meta-analysis (Figure 1). Of the 14 included studies, 1,537 patients were diagnosed with primary hypertension (769 cases in the experimental group and 768 cases in the control group) and used in this meta-analysis. The intervention used for patients is TGD combined with nifedipine in the experimental group and only nifedipine in the control group. The treatment for primary hypertension in the included studies was slightly different; namely, in the TGD combined with nifedipine treatments, the studies used nifedipine sustained-release tablets or nifedipine controlled-release tablets. In addition, the doses of TGD ranged from approximately 93.4 to 180 g, and the dosage of nifedipine for patients ranged from 20 to 60 mg/day by oral administration (Table 2).

### 3.2. Quality of Included Trials

**Figure 2:**
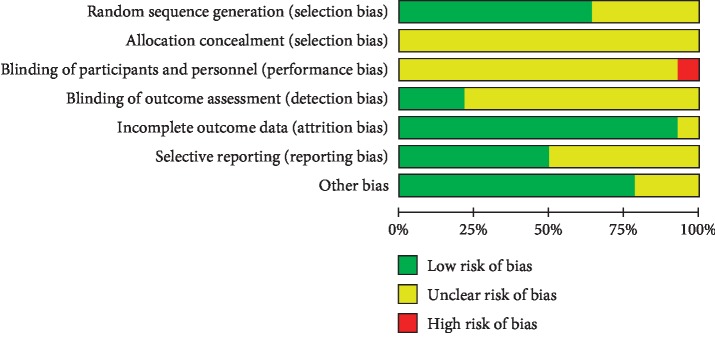
Risk of bias assessment in eligible studies. The quality assessment was conducted by Review Manager 5.3 according to the Cochrane Handbook for Systematic Reviews of Interventions version 5.1.0. Red, high risk of bias; green, low risk of bias; yellow, unclear risk of bias.

All trials were RCTs of participants according to Cochrane risk of bias estimation. The appropriate generation of random distribution sequence was depicted in six [17–19, 24–26] articles. Particular information on distribution was absent from most articles. All studies not used blinding of participants and consequence assessment. Nine [17–21, 23–26] articles had integral outcome data with a low risk of attrition bias. Eight articles [17–19, 21, 23–26] had a low risk of reporting bias as detailed results were given (Figure 2).

### 3.3. Antihypertensive Efficacy (14 Studies)

**Figure 3:**
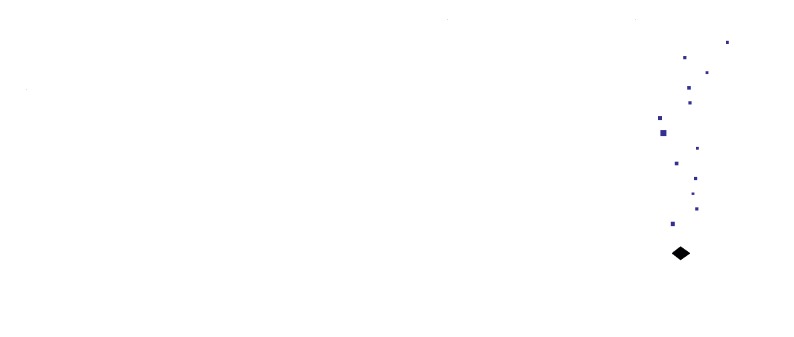
Forest plot of antihypertensive effect. Note: experiment: Tianma Gouteng decoction combined with nifedipine; control: nifedipine.

Effectiveness was defined as an improvement of symptoms. Fourteen articles [15–20, 23–29] reported the total efficacy rate. A fixed-effects model was performed to analyze these studies, and the results showed that TGD combined with nifedipine significantly improved primary hypertension (RR = 1.17, 95% CI: 1.12 to 1.22, and *P* < 0.00001; Figure 3). There was no statistically significant heterogeneity among the individual trials (*P*=0.22, *χ*^2^ = 15.40, and *I*^2^ = 22%).

### 3.4. TCM Improvement (Eight Studies)

**Figure 4:**
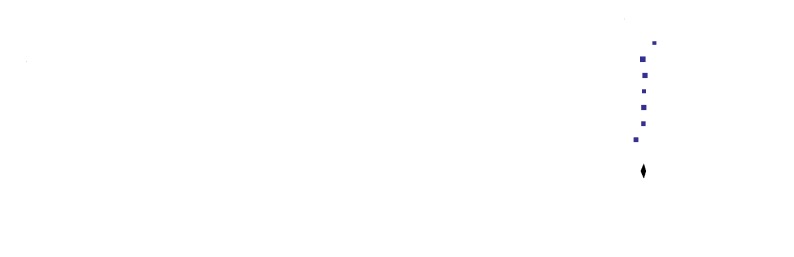
Forest plot of the improvement of traditional Chinese medicine symptoms. Note: experiment: Tianma Gouteng decoction combined with nifedipine; control: nifedipine.

Eight [16–18, 20, 22, 24, 27] studies measured the improvement of TCMs. There was a statistically significant degree of heterogeneity among individual studies (*χ*^2^ = 10.65, *I*^2^ = 44%, and *P*=0.10); therefore, a fixed-effects model was performed for a meta-analysis, which showed that TGD combined with nifedipine can significantly improve TCMs (RR = 1.26, 95% CI: 1.17 to 1.36, and *P* < 0.00001; Figure 4).

### 3.5. Decreasing DBP Effect (Eight Studies)

**Figure 5:**
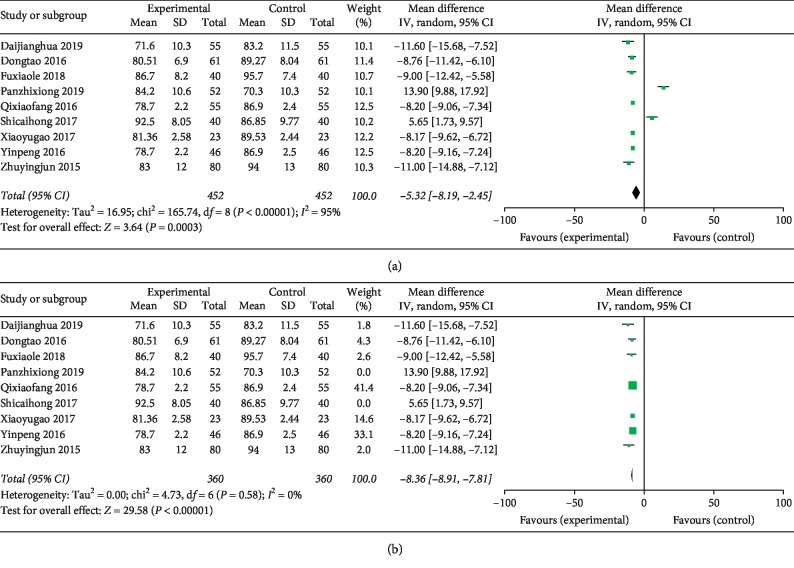
Forest plot of decreasing diastolic blood pressure effect. Note: experiment: Tianma Gouteng decoction combined with nifedipine; control: nifedipine. (a) No sensitivity analysis and (b) after sensitivity analysis.

Eight [17, 21–28] studies investigated the effectiveness of the combination of TGD and nifedipine in reducing DBP. In the meta-analysis, DBP was significantly reduced (MD = −5.32, 95% CI: −8.19 to −2.45, and *P* < 0.00001; Figure 5(a)) with significant heterogeneity among the studies (*χ*^2^ = 165.74, *P*=0.0003, and *I*^2^ = 95%). TGD combined with nifedipine is preferable to nifedipine in reducing DBP of patients. Due to the large heterogeneity, sensitivity analysis was performed by removing 2 studies and recalculated the combined estimate on remaining studies. And the results of DBP showed no heterogeneity (*I*^2^ = 0, MD = −8.36, 95% CI: −8.91 to −7.81, and *P* < 0.00001; Figure 5(b)) when two studies (panzhixiong 2019 and shicaihong 2017) were removed.

### 3.6. Decreasing SBP Effect (Eight Studies)

**Figure 6:**
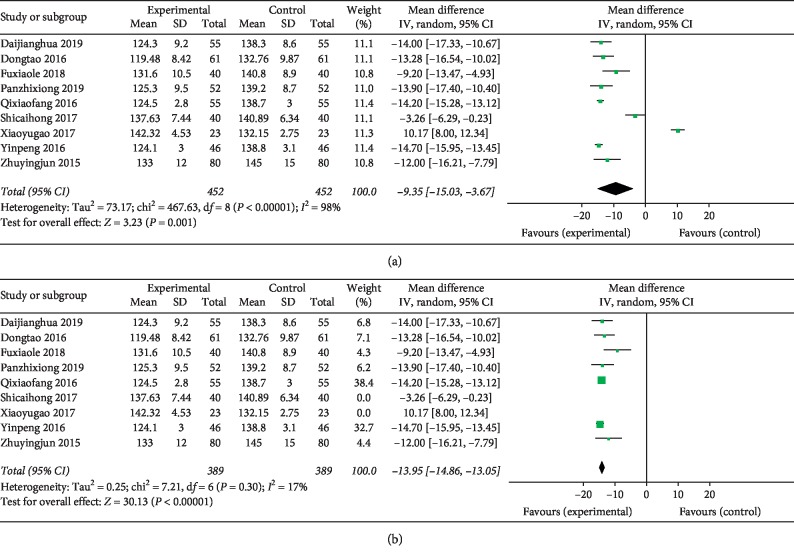
Forest plot for decreasing systolic blood pressure effect. Note: experiment: Tianma Gouteng decoction combined with nifedipine; control: nifedipine. (a) No sensitivity analysis and (b) after sensitivity analysis.

Eight [17, 21–28] trials reported the intervention reflecting SBP. There was heterogeneity among the studies (*χ*^2^ = 467.63, *P* < 0.00001, and *I*^2^ = 98%) and the random-effects model was performed for this analysis. The MD and 95% CI (MD = −9.35, 95% CI: −15.03 to −3.67, and *P*=0.001; Figure 6(a)) indicated a significant decrease of SBP in the experimental group compared with the control group. Sensitivity analysis was performed by removing 2 studies and recalculated the combined estimate on remaining studies due to greater heterogeneity. The result showed a small heterogeneity (*I*^2^ = 17%, MD = −13.95, 95% CI: 14.86 to −13.05, and *P* < 0.00001; Figure 6(b)) when two studies (shicaihong 2017 and xiaoyugao 2017) were removed.

### 3.7. Serum Creatinine (Scr) and Blood Urea Nitrogen (BUN) (One Study)

One trial [17] reported serum creatinine from 90.82 ± 9.47 *μ*mol/L to 70.46 ± 7.51 *μ*mol/L and blood urea nitrogen from 6.91 ± 1.28 mmol/L to 4.17 ± 1.02 mmol/L after treatment in the experimental group. In the control group, the level of serum creatinine reduced from 91.76 ± 10.73 *μ*mol/L to 83.15 ± 8.92 *μ*mol/L and blood urea nitrogen decreased from 6.73 ± 1.35 mmol/L to 5.38 ± 1.87 mmol/L. Compared to the control group, the Scr and BUN level of the experimental group showed a greater decrease. However, the small sample size prevents any firm conclusions from being inferred.

### 3.8. AEs (Two Studies)

Four trials [22–25] provided descriptions on AEs such as dizziness, gastrointestinal reaction, stomach discomfort, diarrhea, and ankle edema. Nevertheless, these symptoms disappeared without treatment.

### 3.9. Publication Bias

**Figure 7:**
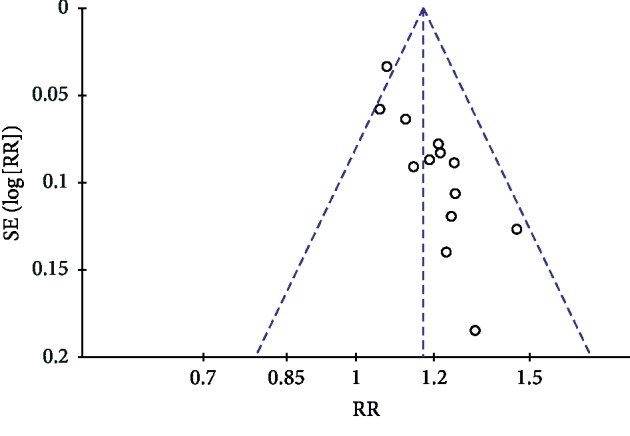
Funnel plot of antihypertensive efficacy for the publication bias.

**Figure 8:**
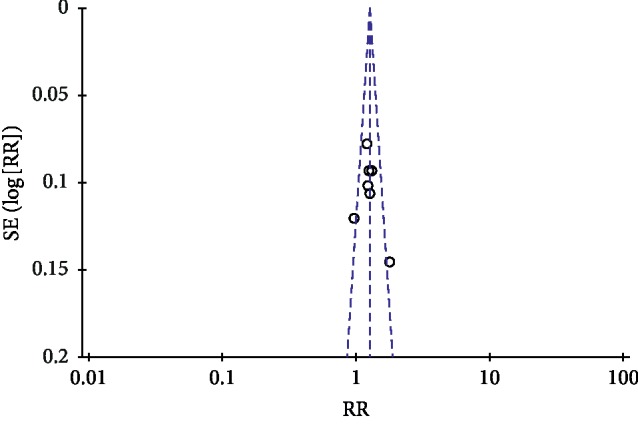
Funnel plot of traditional Chinese medicine symptoms for the publication bias.

**Figure 9:**
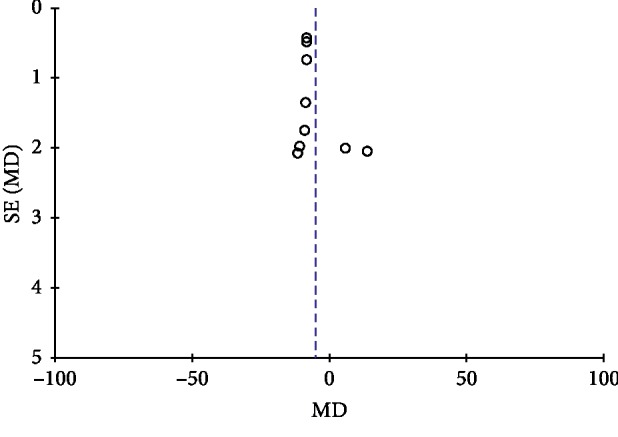
Funnel plot of diastolic blood pressure for the publication bias.

**Figure 10:**
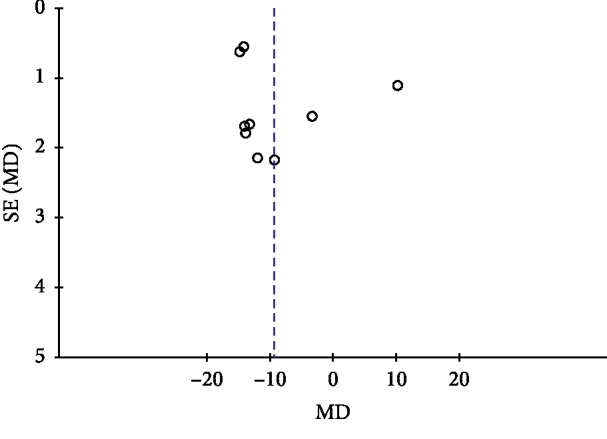
Funnel plot of systolic blood pressure for the publication bias.

Publication bias was conducted by a funnel plot. In this study, funnel plots were conducted of TGD combined with nifedipine vs. nifedipine alone on antihypertensive efficacy, TCMs, and BP. Except for Figure 7, other funnel plots were usually symmetrical, which indicated no evident publication bias (Figures 7–10). Although we conducted comprehensive searches, we identified and included 14 trials; all of them were conducted and published in Chinese. All of the trials had small sample sizes. We tried to avoid language bias and location bias, but we could not exclude potential dissemination bias. Study publications provided only limited descriptions of study design, allocation concealment, and baseline data. All of the RCTs included in this review showed a mostly unclear risk of bias in more than one “risk of bias” domains.

## 4. Discussion

As the first inducer for CVD, hypertension will be the number one “killer” of human beings by 2020 [30, 31]. The overall prevalence of hypertension in adults is approximately 30–45% [32], and a SBP of 20 mmHg higher than average and DBP 10 mmHg higher than average were each associated with a doubling in the risk of death from stroke, heart disease, or other vascular diseases [33]. In a World Health Organization report, the number of hypertensive patients increased from 600 million in 1980 to 1 billion in 2008 [30], and this number is still rising. However, if hypertension is controlled, CVD events will significantly decrease. In the United Kingdom, the incidence of stroke is forecasted to drop by 28%–44%, and the incidence of ischemic heart disease will reduce by 20–35% through the control hypertension [34]. A report from the American College of Cardiology/American Heart Association indicated that there are more potentially preventable CVD events attributable to elevated BP in individuals with higher than with lower risk of CVD [33]. Therefore, treating hypertension is critical for protecting against the occurrence of CVD.

Nifedipine is commonly used to treat hypertension in clinical practice, but it has some limitations [35–39]. In the theory of TCM, the mechanism of primary hypertension pertains to dizziness, headache, hyper-yang of liver, and insufficiency of liver-yin [40]. TGD can treat hyper-yang of liver, as well as the upward disturbance of liver wind [7]. Although there are some limitations for TCM due to the shortage of sufficient studies, increasingly valid evidence-based practice makes it an attractive therapy system for various diseases. Numerous recent studies have found that TGD can affect the renin-angiotensin-aldosterone system to reduce angiotensin and plasma endothelium levels that can influence BP [41]. Sixty-seven hypothalamic protein expressions increased more than two times, and 19 hypothalamic protein expressions decreased more than two times after taking TGD. These changes may be related to the mechanism of TGD in treating hypertension [42]. TGD can improve endothelial function in patients with hypertension via the mechanism of increasing GCH-PX and CAT content, remove excess oxygen free radicals, and prevent lipid peroxidation of vascular endothelial cells [43]. TGD can reduce vasoconstrictor substances in hypertensive patients and protect and regulate vascular endothelium secretory function [44]. Moreover, as a primary component of TGD prescription, *Gastrodia elata* plays an important role in regulating the flow of coronary blood in patients [45]. *Gastrodia elata* could perform its vasodilator effect not only by inhibiting vascular smooth muscle contraction, but also by enhancing blood vessel elasticity and stabilizing the arterial structure [46]. *Gastrodia elata* can antagonize epinephrine, regulate blood vessels, and dilate small blood vessels, thereby regulating blood pressure. In addition, several clinical studies found that *Uncaria* could decrease BP effectively by regulating the above biomarkers and metabolic pathways [47]. The antihypertensive effect of *Uncaria* alkaloids is related to the decrease in frequency [48].

In clinical studies, total efficiency is a measure for judging the antihypertensive efficacy of drugs. As the results of this meta-analysis showed, the total effective rate for the treatment of hypertension in the experimental group was 90.93% (652/717), which was higher than the control group (77.79% (557/716)). Compared with the control group, the experimental group showed better in antihypertension effect (RR = 1.17, 95% CI: 1.12 to 1.22, and *P* < 0.00001). Hypertension in patients is also accompanied by dizziness, headache, tinnitus, and insomnia [40]. However, these symptoms significantly improved in the experimental group (RR = 1.26, 95% CI = 1.17 to 1.36, and *P* < 0.00001). The BP level also reduced in the experimental group more than the control group (DBP : MD = −5.32, 95% CI: −8.19 to −2.45, and *P* < 0.00001; SBP : MD = −9.35, 95% CI: 15.03 to −3.67, and *P*=0.001). Only two studies reported AEs of TGD combined with nifedipine.

However, substantial heterogeneity was detected between included studies when we studied SBP and DBP outcomes. First, a sensitivity analysis was conducted, in which 1 study at a time was removed and the individual study would not have a significant impact on the results. Second, we conducted a sensitivity analysis by removing 2 studies and recalculated the combined estimate on remaining studies. The results of SBP showed a small heterogeneity (*I*^2^ = 17%, MD = −13.95, 95% CI: 14.86 to −13.05, and *P* < 0.00001) when two studies (shicaihong 2017 and xiaoyugao 2017) were removed. And the results of DBP showed no heterogeneity (*I*^2^ = 0, MD = −8.36, 95% CI: −8.91 to −7.81, and *P* < 0.00001) when two studies (panzhixiong 2019 and shicaihong 2017) were removed. We made a detailed analysis of the included literature and found that there were significant differences in the sex ratio of patients in the experimental group in the shicaihong 2017 and xiaoyugao 2017 studies. Study publications provided only limited descriptions of study design, allocation concealment, and baseline data, and there are a few indicators of measurement. All of the RCTs included in this review showed a mostly unclear risk of bias in more than one “risk of bias” domains. These reasons may lead to poor heterogeneity in the research process, as well as funnel diagram asymmetry. It is suggested that international standards should be used in clinical research to improve the quality of methodology and strengthen the quality of research and optimization methodology. At the same time, the details such as the generation of random sequence, the concealment of distribution, and the implementation of random allocation should be clarified in the research report. In addition, it should also develop in the direction of international cooperation, multicenter, large sample, complex random grouping, and so on. In addition, there are limitations to this research, such as the low quality of eligible trials, the lack of strict methodologies, and the employment of sole race rather than a more varied population sample. It is necessary to examine the results using other rigorous and large-scale RCTs.

## 5. Conclusion

According to the results and conclusions of the article, we can see that the combination of TGD and nifedipine has a better effect in the treatment of hypertension, so we suggest that we can adopt the method of combination of traditional Chinese and Western medicine according to the patient's condition. However, our findings must be handled with care because of the small sample size and low quality of clinic trials cited. Other rigorous and large-scale RCTs are in need to confirm these results.

## Figures and Tables

**Table 1:** Basic characteristics of the included trials.

Study	Patients (*n*)	Sex (male/female)		Diagnostic standard
	Experimental group/control group	Experimental group	Control group	
Peifeng, 2015 [15]	33/33	21/12	C:21/12	NR
Xiaofei et al., 2016 [16]	40/40	22/18	20/20	GPTH2010 and GPCREDTCM
Tao, 2016 [17]	61/61	22/18	20/20	GPTH2010 and CMDDES
Yingke, 2014 [18]	60/60	32/28	33/27	GPCREDTCM
Jinbo, 2015 [19]	154/153	87/67	75/78	NR
Fan and Lili, 2015 [20]	30/30	NR	NR	CMDTHDESN
Pan, 2019 [21]	52/52	26/26	25/27	NR
Xiaofang, 2016 [22]	55/55	30/25	32/23	GPTH2010
Fu, 2018 [23]	40/40	22/18	24/16	GPTH2010
Caihong, 2017 [24]	40/40	5/15	23/17	GPTH2010 and GPCREDTCM
Dai, 2019 [25]	55/55	NR	NR	NR
Yugao, 2017 [26]	23/23	14/9	13/10	GPCREDTCM
Peng et al., 2016 [27]	46/46	25/21	26/20	NR
Yingjun, 2015 [28]	80/80	48/32	46/34	GPTH2010 and GPCREDTCM

Note: NR: no report; GPTH2010: Guide to Prevention and Treatment of Hypertension 2010; GPCREDTCM: Guiding Principles for Clinical Research of New Drugs in Traditional Chinese Medicine; CMDDES: Chinese Medicine Dialectical Diagnosis Efficacy Standard; CMDTHDESN: Chinese Medicine Diagnosis and Treatment of Heart Disease Efficacy Standards and Norms; GPTHC: Guide to Prevention and Treatment of Hypertension in China (trial version).

**Table 2:** Intervention characteristics of the included trials.

Study	Dosage		Duration	Outcome
	Control group	Trial group		
Peifeng, 2015 [15]	NF (2 times/d, 30 mg/times)	TGD (52.5 g/times, 2∼3 times/d) + NF (2 times/d, 30 mg/times)	1 month	Ae
Xiaofei et al., 2016 [16]	NF (1 times/d, 30 mg/times)	TGD (51.2 g/times, 2∼3 times/d) + NF (1 times/d, 30 mg/times)	2 weeks	Ae TCMS
Tao, 2016 [17]	NF (2 times/d, 10 mg/times)	TGD (48.4 g/times, 2∼3 times/d) + NF (2 times/d, 10 mg/times)	8 weeks	BP Scr BUN
Yingke, 2014 [18]	NFS (2 times/d, 10 mg/times)	TGD (52 g/times, 2∼3 times/d) + NFS (2 times/d, 10 mg/times)	2 weeks	Ae TCMS
Jinbo, 2015 [19]	NFS (2 times/d, 10 mg/times)	TGD (57 g/times, 2 times/d) + NFS (2 times/d, 10 mg/times)	2 weeks	Ae
Fan and Lili, 2015 [20]	NFS (1 times/d, 5 mg/times)	TGD (87.5 g/times 2 times/d) + NFS (1 times/d, 5 mg/times)	3 months	Ae
Pan, 2019 [21]	NFS (1 times/d, 20 mg/times)	TGD (90 g/times, 2 times/d) + NFS (1 times/d, 20 mg/times)	2 weeks	BP
Xiaofang, 2016 [22]	NFS (2 times/d, 20 mg/times)	TGD (125 g/d) + NFS (2 times/d, 20 mg/times)	NR	Ae BP Ar
Fu, 2018 [23]	NFS (2 times/d, 20 mg/times)	TGD (57 g/times, 2 times/d) + NFS (2 times/d, 20 mg/times)	6 months	Ae BP Ar
Caihong, 2017 [24]	NFS (1 times/d, 30 mg/times)	TGD (79 g/times, 2 times/d) + NFS (1 times/d, 30 mg/times)	NR	Ae BP
Dai, 2019 [25]	NF (1 times/d, 20 mg/times)	TGD (NR) + NF (1 times/d, 20 mg/times)	2 weeks	Ae BP Ar
Yugao, 2017 [26]	NFS (2 times/d, 10 mg/times)	TGD (52 g/times, 3 times/d) + NFS (2 times/d, 10 mg/times)	8 weeks	Ae BP life quality
Peng et al., 2016 [27]	NFC (1 times/d, 30 mg/times, after half a month, 2 times/d, 30 mg/times)	TGD (46.7 g/times 2 times/d) + NFC (1 times/d, 30 mg/times, after half a month, 2 times/d, 30 mg/times)	2 months	Ae BP
Yingjun, 2015 [28]	NFS (2 times/d, 10 mg/times)	TGD (68.8 g/times, 3 times/d) + NFS (2 times/d, 10 mg/times)	NR	Ae BP

Notes: NF: nifedipine; NFS: nifedipine sustained-release tablets; NFC: nifedipine controlled-release tablets; NR: no report; Ae: antihypertensive effect; TCMS: traditional Chinese medicine symptoms; BP: blood pressure; Scr: serum creatinine; BUN: blood urea nitrogen; Ar: adverse reactions. TGD was taken as a decoction when taken by patients.

## Data Availability

All data generated or analyzed during this study are included in this article.

## Abbreviations:

CVD: Cardiovascular disease
TGD: Tianma Gouteng decoction
RCTs: Randomized controlled trials
BP: Blood pressure
DBP: Diastolic blood pressure
SBP: Systolic blood pressure
TCMs: Traditional Chinese medicine symptoms
RR: Risk ratio
OR: Odds ratio
MD: Mean difference
CI: Confidence intervals
Scr: Serum creatinine
BUN: Blood urea nitrogen.
